# Oxidative Stress Decreases Functional Airway Mannose Binding Lectin in COPD

**DOI:** 10.1371/journal.pone.0098571

**Published:** 2014-06-05

**Authors:** Hai B. Tran, Jessica Ahern, Greg Hodge, Phillip Holt, Melinda M. Dean, Paul N. Reynolds, Sandra Hodge

**Affiliations:** 1 Lung Research, Hanson Institute and Department Thoracic Medicine, Royal Adelaide Hospital, Adelaide, South Australia, Australia; 2 Department of Medicine, University of Adelaide, Adelaide, South Australia, Australia; 3 Research and Development, Australian Red Cross Blood Service, Brisbane, Queensland, Australia; University of Bern, Switzerland.

## Abstract

We have previously established that a defect in the ability of alveolar macrophages (AM) to phagocytose apoptotic cells (efferocytosis) and pathogens is a potential therapeutic target in COPD. We further showed that levels of mannose binding lectin (MBL; required for effective macrophage phagocytic function) were reduced in the airways but not circulation of COPD patients. We hypothesized that increased oxidative stress in the airway could be a cause for such disturbances. We therefore studied the effects of oxidation on the structure of the MBL molecule and its functional interactions with macrophages. Oligomeric structure of plasma derived MBL (pdMBL) before and after oxidation (oxMBL) with 2,2′-azobis(2-methylpropionamidine)dihydrochroride (AAPH) was investigated by blue native PAGE. Macrophage function in the presence of pd/oxMBL was assessed by measuring efferocytosis, phagocytosis of non-typeable *Haemophilus influenzae* (NTHi) and expression of macrophage scavenger receptors. Oxidation disrupted higher order MBL oligomers. This was associated with changed macrophage function evident by a significantly reduced capacity to phagocytose apoptotic cells and NTHi in the presence of oxMBL vs pdMBL (eg, NTHi by 55.9 and 27.0% respectively). Interestingly, oxidation of MBL significantly reduced macrophage phagocytic ability to below control levels. Flow cytometry and immunofluorescence revealed a significant increase in expression of macrophage scavenger receptor (SRA1) in the presence of pdMBL that was abrogated in the presence of oxMBL. We show the pulmonary macrophage dysfunction in COPD may at least partially result from an oxidative stress-induced effect on MBL, and identify a further potential therapeutic strategy for this debilitating disease.

## Introduction

Chronic obstructive pulmonary disease (COPD), a lung disease mostly associated with cigarette smoking, is predicted to be the third leading cause of death in the world by 2020 [1]. COPD is incurable, existing treatments are largely symptomatic, and there is an urgent need for identification of new therapies. Macrophages are the prominent leucocyte found in lungs. We have shown that a dysfunction of pulmonary macrophages in COPD may provide a novel target for therapeutic manipulation [2]–[4]. In particular, a significant defect in the ability of pulmonary macrophages to phagocytose apoptotic epithelial cells (defective efferocytosis) established in the airways of COPD patients may contribute to an excess of apoptotic material, secondary necrosis and perpetuation of chronic inflammation [5]. We have also demonstrated impaired phagocytosis of bacteria in COPD; an important finding given the increased bacterial colonization and increased susceptibility to infectious exacerbations in these patients [6], [7]. These defects are not isolated to COPD, as we have also reported increased numbers of apoptotic bronchial epithelial cells and defective efferocytosis in the airways in other chronic lung diseases including severe asthma and chronic lung transplant rejection [8], [9]. Importantly, these defects could be substantially overcome using macrophage-targeted therapies [6], [10]–[14]. In a human phase II study of COPD subjects we found a significant improvement in the ability of alveolar macrophages to phagocytose apoptotic airway epithelial cells and bacteria following treatment with low-dose azithromycin [6], [10], [11]. Subsequently, Nakanishi et al showed that administration of clarithromycin prevented the onset of emphysema in smoke-exposed mice [15]. Although these results are exciting and establish the important biological paradigm that macrophage function can be manipulated for therapeutic gain, the use of long-term antibiotic therapy has obvious drawbacks including the potential emergence of antibiotic-resistant strains of bacteria.

Mannose binding lectin (MBL) is a key component of the innate immune system produced mostly by hepatocytes for secretion into circulation. MBL belongs to the family of collectins, which includes also C1q and lung surfactant proteins. As a soluble pattern recognition receptor MBL is involved in phagocytosis of bacteria as well as recognition and clearance of apoptotic cell bodies [16], [17]. Presence of MBL in bronchoalveolar lavage (BAL) of children with airway infections but not controls suggests its role in pulmonary defence [18]. We have extensively investigated MBL in lung diseases, in particular in smokers and COPD patients and in a smoke-exposed mouse model [8], [12]. Our data showed significantly reduced levels of functional MBL in the airway of COPD subjects and healthy smokers compared to non-COPD controls, and further demonstrated a positive correlation between efferocytosis and MBL in the airway [8]. Nebulised administration of plasma derived MBL (pdMBL) significantly restored alveolar macrophage and lung tissue macrophage efferocytic ability and reduced airway inflammation in smoke-exposed mice *in vivo* [12]. These findings strongly support the further study of pdMBL as a novel macrophage-targeted therapy in COPD. It remains however to be determined why levels of MBL are low in the airways of COPD patients while levels in plasma are normal, suggesting a specific airway-associated effect on MBL.

The molecular structure of MBL includes a basic subunit consisting of three peptide chains (homotrimer); such homotrimers can assemble into several oligomeric forms from dimers to hexamers, thus representing its quaternary structure. It is the higher order oligomers which are thought to be essential for the functions of MBL [19]. The quaternary structure may also provide protection of MBL from susceptibility to metalloproteinase proteolysis [20]. Given the high levels of oxidative stress that we and others have reported in COPD/smoker airways [13], [21], oxidative modifications could be hypothesized to disrupt the quaternary structure of MBL, thus making the molecule both vulnerable to proteolysis and less efficient in its functional capacity. This study presents data demonstrating that treatment of native MBL with a synthetic oxidizing agent led to both disruption of its higher order oligomeric structures and functional impairment, assessed by induction of macrophage scavenger receptor A1 (SRA), stimulation of phagocytosis of NTHi and efferocytosis of apoptotic bronchial epithelial cells by macrophages.

## Methods

### Antibodies and Reagents

For efferocytosis and phagocytosis assay: CD13 phycoerythrin cyanine-7 (PE-Cy7) (BD Biosciences, San Jose CA, USA), sytox orange and sytox green (Molecular Probes, Oregon, USA) were employed. For other flow cytometry staining: mannose receptor phycoerythrin (PE) (Immunotech/Coulter, Marseille, France); Colec-12 unconjugated (R&D Systems, Minneapolis, MN, USA), detected with anti-goat IgG allophycocyanin (APC) (R&D) and, CD204 PE (R&D) were employed. For immunofluorescence: MSR1/SRA unconjugated rabbit polyclonal antibody (Abcam, Cambridge, UK and sheep anti-rabbit IgG-Cy3, F(ab’)2 fragment (Sigma-Aldrich, Saint Louis, MO, USA) was employed. All other reagents, if not otherwise specified, were from Sigma-Aldrich (Saint Louis, MO, USA).

### Bronchoscopy Sampling and Subject Selection

BAL was obtained via flexible bronchoscopy from volunteers as we have previously reported [2]–[4]. Ethical approval was granted by the Royal Adelaide Hospital Ethics Committee and written informed consent was obtained for each donor of BAL. For *in vitro* studies, BAL was collected from 10 healthy adult volunteers with no history of asthma or allergy and normal lung function.

### Preparation of BAL and Purification of Alveolar Macrophages

BAL was prepared, total cells and macrophages counted and macrophages purified by adhesion to plastic as previously described [2]–[4]. Briefly, for assessment of phagocytosis/efferocytosis and scavenger receptor expression, BAL cells resuspended in RPMI 1640 media (Gibco, Karlsruhe, Germany) supplemented with 10% foetal calf serum and 1% penicillin/gentamicin (Gibco) (hereinafter referred to as R10) were plated at 2×10^5^ cells/well on 24-well plates and incubated at 37°C/5% CO_2_ for 2 h. The plates were tapped to loosen non-adhered cells which were then gently removed with the fluid. Alveolar macrophages adhered to the wells were then replenished with fresh R10 medium for further experiments.

### Preparation of Differentiated THP-1 Macrophages

THP-1 monocytic cells (American Type Culture Collection, Manassas, VA, USA) growing in continuous culture were differentiated into macrophages following phorbol myristate acetate (PMA) stimulation for 72 h as previously reported [4], and cells treated as described for alveolar macrophages.

### Oxidative Modification of Plasma Derived MBL

Affinity purified plasma derived MBL (pdMBL) was obtained from pooled human plasma as described earlier [12]. Stock of pdMBL (1 mg/mL in Tris Buffered Saline, TBS) was incubated with a final concentration of 74 mM of the synthetic oxidizing agent 2,2′-azobis(2-methylpropionamidine) dihydrochloride (AAPH) (Novachem Pty Ltd, Collingwood, VIC, Australia) for 2 h at 37°C.

### Measuring Oxidative Changes to MBL Structure

Samples of pdMBL and its oxidized form (oxMBL) were analysed by the Adelaide Proteomics Centre, University of Adelaide, South Australia. Blue native electrophoresis was applied according to a published protocol [22]. Briefly, equal amounts of pdMBL and oxMBL (10 ug protein per well) were run on 4–12% gradient polyacrylamide gel electrophoresis (PAGE) in native conditions to determine their oligomeric state. The protein bands were visualized by Blum’s silver stain [22].

### Treatment of Macrophages with pdMBL and oxMBL

Adhered macrophages were incubated for 18 h at 37°C/5% CO_2_ in R10 medium containing 20 µg/mL of pdMBL or oxMBL or R10 only (control).

### Efferocytosis of Apoptotic Bronchial Epithelial Cells by Alveolar and THP-1 Macrophages

Efferocytosis was assessed as previously reported [2], [3] with minor modifications. A final concentration of 20 µg/mL pdMBL or oxMBL was present during the efferocytosis assays, which were performed in triplicate. Briefly, a 16 HBE bronchial epithelial cell line maintained in continuous culture with R10 medium was induced to apoptosis using a 305 nm UV source (UVP, Upland, CA, USA), then labelled with 250 nM sytox orange. The supernatant from each macrophage culture on the 24-well plates was removed and 500 uL of sytox orange-labelled apoptotic 16 HBE cells added to each well at a 10∶1 ratio. Cells were further incubated at 37°C/5% CO_2_ for 1.5 hr then supernatant removed. Following a 5 min incubation with 500 uL ice-cold phosphate buffered saline (PBS), cells were lifted by gentle continuous pipetting then transferred to FACS tubes, pelleted by centrifugation, then stained with 3 µL of CD13 PE-Cy7 for 10 min. Following a wash with 2 mL of Isoflow (Immunotech/Coulter) supplemented with 0.5% bovine serum albumin (hereinafter referred to as wash buffer), cells were pelleted again, resuspended in 100 µL of wash buffer, 1 mg/mL of trypan blue was added to quench autofluorescence and 30,000 total events per tube were acquired immediately using a FACSCanto II Flow Cytometer (BD).

### Phagocytosis of NTHi by AM and THP-1 Macrophages

NTHi originally isolated from the sputum of an adult with pneumonia was kindly provided by Susan Pizzutto, Menzies School of Health Research, Darwin, Northern Territory, Australia. The sample was obtained with written consent according to an ethics approval granted by the Human Research Ethics Committee of the Northern Territory Department of Health and Menzies School of Health Research. Frozen stock of NTHi was defrosted on ice, then 1.36×10^8^ CFU of NTHi were washed twice with ice cold PBS and pelleted by centrifugation. NTHi were then permeabilized in 400 µL of 70% ethanol at room temperature for 30 min, then washed twice with 0.85% saline and pelleted by centrifugation at 14,000 rpm/4°C for 5 min. Permeabilized NTHi resuspended in 0.85% saline were stained with 5 uM sytox green at room temperature for 5 min, then washed with wash buffer and pelleted by centrifugation and the concentration adjusted to 4×10^7^ CFU/mL in R10 medium. For the phagocytosis assay, 2×10^7^ CFU of sytox green stained NTHi were added to adhered macrophages pre-treated with pdMBL/oxMBL/control (media, no MBL) in 24-well plates. Cells were incubated at 37°C/5% CO_2_ for 1.5 h, then treated as described for the efferocytosis assay.

### Flow Cytometric Analysis of the Effects of Oxidation on Macrophage Scavenger Receptors

Multi-parameter flow cytometry was used to assess surface expression of scavenger receptors in macrophages pre-treated with pdMBL/oxMBL/control (media, no MBL) as previously reported [3], [11], [13].

### Immunofluorescence Analysis of the Effects of Oxidation on Intracellular Macrophage Scavenger Receptor A1

Cytospin preparations of pdMBL/oxMBL/control treated THP-1 macrophages (∼40,000 per spot) were fixed with 2.5% formalin in phosphate-buffered saline (PBS) for 15 min, washed twice with 0.05% Tween in Tris-buffered saline pH 7.5 (TBST), air dried overnight at room temperature, then stored at −20°C. Before immunostaining, cells were permeabilized for 5 min with 0.1% SDS in PBS, rinsed 3 times with TBST, then blocked with a serum-free protein solution (Dako A/S, Glostrup, Denmark) for 1 h at room temperature. Cells were incubated overnight with the primary antibody at 4°C, washed 3 times for 10 min with TBST on a horizontal shaker, incubated with the Cy3-conjugated secondary antibody for 1 h at RT and washed 5 times with TBST as above. For counterstaining of nuclei solution of 4′,6-diamidino-2-phenylindole (DAPI, 200 ng/mL) in PBS was applied for 15 min before washing. Coverslips were then mounted and cells were visualized in a Zeiss microscope equipped with HBO 100 illuminating system, AxioCam MRn digital camera and AxioVision 4.8.1 software (Carl Zeiss GmbH, Goettingen, Germany). For quantitative analysis, microphotos were taken from at least 9 optical fields under 40x objective, with selection of optical fields and focusing carried out in the DAPI channel to prevent bias. Mean fluorescence intensity in the Cy3 channel was measured using the particle analysis function of the ImageJ software (NIH, Bethesda, MD, USA).

### Statistical Analyses

Statistical analysis was performed using non-parametric (Kruskal Wallis plus post hoc Mann Whitney) or the Wilcoxon test for comparisons of paired data. Analyses were performed using SPPS software; *p*<0.05 was considered significant.

## Results

### Oxidative Modification of Plasma Derived MBL

**Figure 1:**
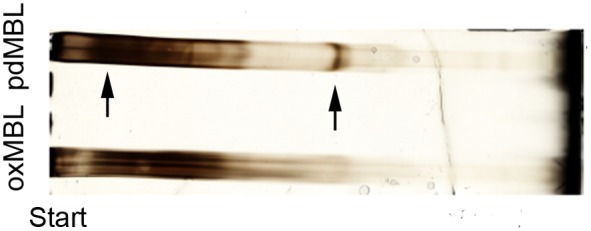
Loss of higher-order quaternary structures in oxMBL vs pdMBL. To mimic high levels of oxidative stress, pdMBL was exposed to 74′-azobis(2-methylpropionamidine)dihydrochloride (AAPH). Polyacrylamide gel electrophoresis was then applied to measure changes to MBL by separating the different MW. Protein samples were loaded 10 ug MBL per lane for blue native PAGE and visualized with silver stain. Figure shows a loss of mid-higher order oligomeric structures in oxMBL vs pdMBL.

Equal amounts of plasma-derived MBL in native state (pdMBL) or oxidized by AAPH (oxMBL) were run on polyacrylamide gel electrophoresis (PAGE) under native conditions to measure changes to oligomer state of MBL. Whereas pdMBL showed both low molecular weight components and prominent bands representing higher order quaternary structures, oxMBL was visualised as only low molecular weight components. Representative results are shown in **Figure 1**.

### Oxidation Abrogates pdMBL-induced Stimulation of Both Efferocytosis of Apoptotic Bronchial Epithelial Cells and Phagocytosis of NTHi by Human Macrophages

**Figure 2:**
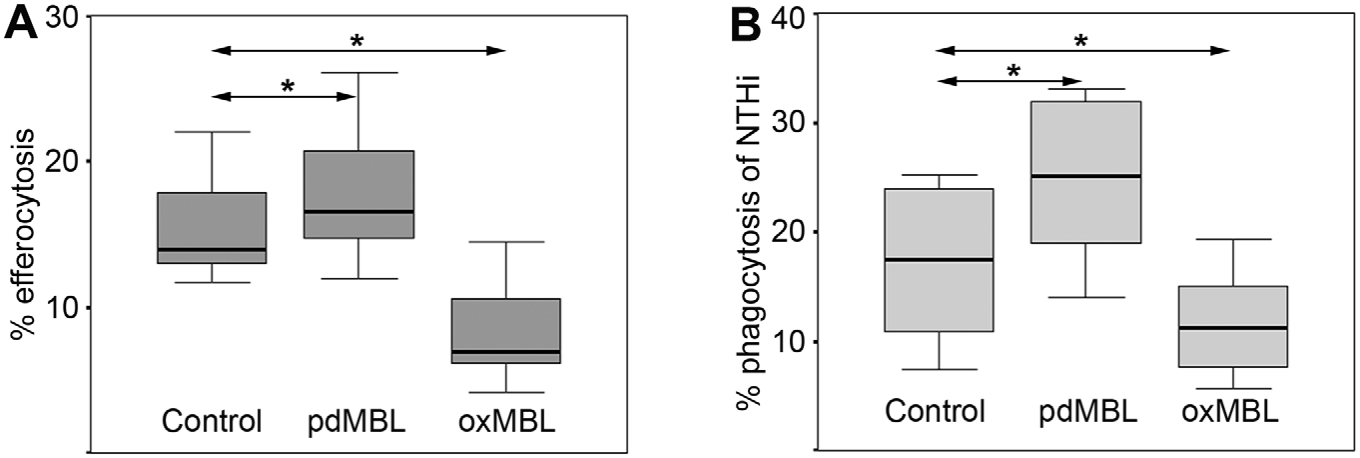
pdMBL but not oxMBL enhanced phagocytosis by human alveolar macrophages. There was a significant increase in phagocytosis of **A**. apoptotic bronchial epithelial cells (*, p<0.05; Wilcoxon; data pooled from 9 experiments.) and **B**. Phagocytosis of NTHi (*, p<0.05; Wilcoxon; data pooled from 12 experiments) in the presence of pdMBL. Oxidation of MBL significantly reduced the phagocytic ability for both apoptotic cells and bacteria vs both control and pdMBL treatment.

The functional capability of pdMBL was firstly assessed in *in vitro* assays of efferocytosis and phagocytosis. Pre-treatment of human alveolar macrophages with pdMBL increased efferocytosis of apoptotic bronchial epithelial cells and phagocytosis of NTHi, a pathogen frequently implicated in exacerbations of COPD (consistent with a previous report of enhanced phagocytosis of *S. aureus* and *E. coli* by pre-incubation of rat Kupffer cells with MBL [23]). oxMBL did not stimulate but rather showed an inhibitory effect on both phagocytosis of the pathogen and efferocytosis of apoptotic cells (Figure 2A, B). Similar results were obtained in further experiments in which macrophages derived from THP-1 monocytes were tested for stimulatory effects of pdMBL and inhibitory effects of oxMBL (data not shown).

### Flow Cytometric Analysis of the Effects of Oxidation on Macrophage Scavenger Receptors

**Figure 3:**
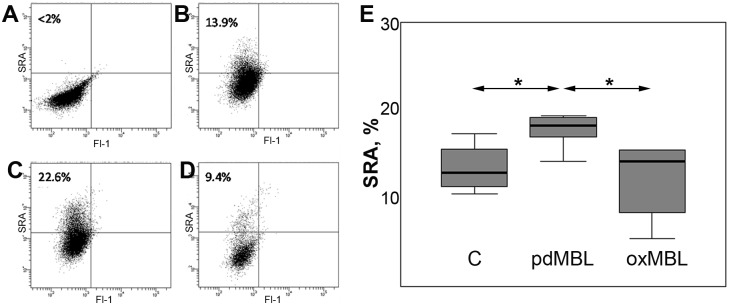
Oxidation negated the stimulatory effects of pdMBL on BAL-derived alveolar macrophage surface expression of SRA detected by flow cytometry. **A–D**. Representative flow cytometry plots showing SRA expression following treatment with pdMBL or oxMBL. Debris, lymphocytes and cell fragments were firstly excluded based on forward- and side-scatter characteristics then macrophages identified by positive staining for CD13 and high autofluorescence (not shown). **A**. Control tube of alveolar macrophages stained with IgG control was included and the staining patterns used to set quadrant markers for flow cytometric analysis of **B**. SRA expression in untreated macrophages (13.9%), **C**. SRA expression in macrophages treated with plasma-derived MBL (22.6%), **D**. SRA expression in macrophages treated with oxidized MBL (9.4%). **E**. There was a significant increase (*, p<0.05; Wilcoxon; data pooled from 7 experiments using BAL-derived alveolar macrophages) in % expression of SRA in the presence of pdMBL. Oxidation of MBL abrogated this stimulation effect.

The increased macrophage phagocytic capacity observed following treatment with pdMBL may be the result of mechanisms involving the up-regulation of pattern recognition receptors involved in phagocytosis or efferocytosis. Of note, scavenger receptor A1 (SRA) has been shown to exhibit increased surface expression in rat Kupffer cells treated with recombinant MBL [23]. To address this hypothesis multi-parameter flow cytometric analysis of scavenger receptors on human alveolar macrophages treated with pdMBL, oxMBL or control (untreated) was performed. No significant changes in surface expression of mannose receptor or Colec12 (SCARA4) were detected (data not shown). Expression of CD204/SRA was significantly increased. There was a significant (p<0.05; Wilcoxon; data pooled from 7 experiments using BAL-derived alveolar macrophages) increase in % expression of SRA on macrophages following stimulation of cells with pdMBL (Figure 3). Oxidation of MBL significantly reduced the expression of SRA vs both control and pdMBL treatment (Figure 3).

### Immunofluorescence Analysis of the Effects of Oxidation on Intracellular Macrophage Scavenger Receptor A1

**Figure 4:**
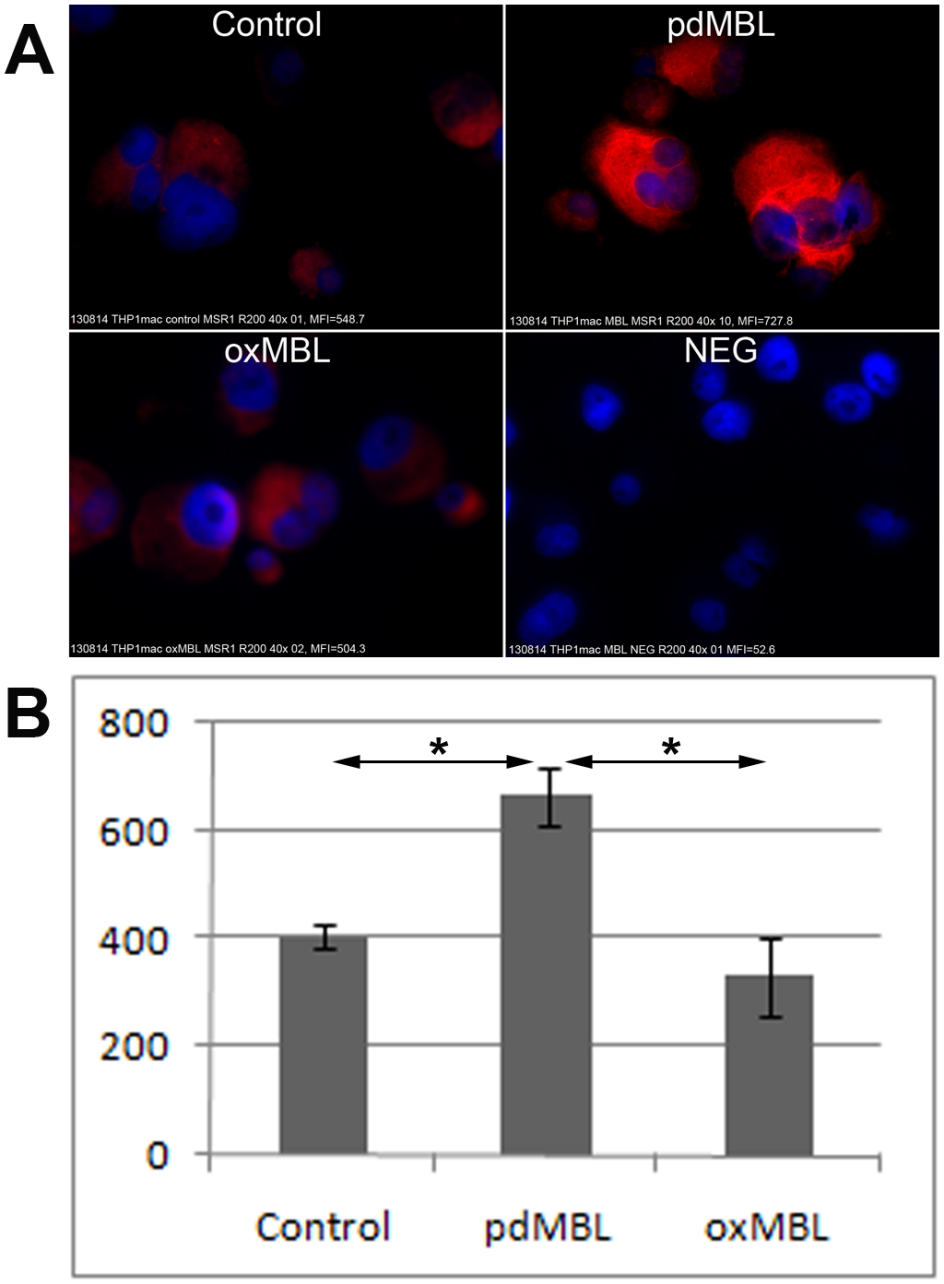
Increase of SRA expression in THP-1 macrophages following stimulation with pdMBL but not oxMBL. **A**. Immunofluorescence staining of SRA (red) in THP-1 macrophages (blue = DAPI). Images were representative of 3 experiments showing similar results. **B**. Quantitative measurement of SRA immunofluorescence in THP-1 macrophages by ImageJ software (MFI±SEM). There was a significant increase (*, p<0.05; Wilcoxon) in immunofluorescence staining of SRA in the presence of pdMBL. Oxidation of MBL abrogated this stimulation effect.

Immunofluorescence staining revealed a strong stimulatory effect of pdMBL on intracellular expression of SRA by THP-1 macrophages, which was significantly negated with application of oxMBL (Figure 4A shows representative images of 3 experiments that showed similar results). Quantitative measurement of SRA immunofluorescence in THP-1 macrophages by ImageJ software demonstrated a significant (p<0.05; Wilcoxon) increase in immunofluorescence staining of SRA in the presence of pdMBL (Figure 4B). Oxidation of MBL significantly reduced the expression of SRA vs both control and pdMBL treatment.

## Discussion

It remains unknown why MBL levels in smokers and COPD patients (and other chronic inflammatory lung diseases) are reduced in the airways though remain unchanged in the circulation [8]. A likely reason could be an increased catabolism *in situ* in conditions of chronic inflammation. In particular, neutrophilic release/activation of proteolytic enzymes is implicated as a key destructive mechanism in COPD [24]. Of the primary sequence of MBL, the collagen-like domain of MBL has been identified as susceptible to proteolysis by MMPs, and importantly, the quaternary structure may protect it from enzymatic attacks [20]. Oxidative damage has been earlier documented for lung proteins of the collectin family such as surfactant protein (SP-) A [25], [26] and SP-D [27]; of note significant changes of quaternary structure were also documented for SP-D in oxidative conditions both *in vitro* and *in vivo* [27]. PdMBL comprises of several oligomeric forms of which the higher molecular weight forms are biologically active [19]. Although reducing gels could not be used for analysis of such forms because of possible breaking of disulfide bonds which stabilize the quaternary structure, the native gel electrophoresis revealed a change induced by oxidation in the mid-to-high molecular weight range, suggesting that oxidative damage to MBL involves disruption of its higher order quaternary structure. In oxidative conditions of the airways of smokers and COPD patients, this may initiate enzymatic breakdown of MBL by making the collagen-like domain accessible for MMPs attack.

The functional capability of MBL is reported to be dependent on its oligomerization state. Genetic mutations responsible for MBL functional deficiency have long been mapped to amino acid substitutions in the collagen-like domain which lead to impairment of the protein oligomerization [28], [29]. In line with this concept and with our findings in previous studies into the pathogenesis of COPD, here we showed that an oxidative damage involving a loss of higher order quaternary structure of pdMBL could also lead to a loss of its functional capability in stimulation of human alveolar macrophages to phagocytose NTHi or efferocytosis of apoptotic bronchial epithelial cells. Quantitative analysis of phagocytosis and efferocytosis in macrophages post treatment with oxidized pdMBL suggests there was not merely a loss of function but an inhibitory effect. This raises the very interesting question which requires further studies to address, that oxidized proteins in the COPD lung could inhibit efferocytosis and phagocytosis by acting as competitive ligands to scavenger receptors. Although traditionally recognized as a mediator of macrophage phagocytosis of pathogens, MBL is now recognized to regulate the clearance of apoptotic cells [16], [17]. There are several reported macrophage receptors for MBL that are involved in efferocytosis, including the CD91-calreticulin complex [16] and CR35/CR1 [30]. We have previously shown that alveolar macrophages from current-smoker COPD subjects exhibited reduced expression of several of the receptors involved in efferocytosis including CD31, CD91 and hyaluron receptor, compared to healthy never-smoker controls [3]. However, given our findings of defects in phagocytosis of both apoptotic cells and bacteria in COPD, we focussed our current investigations on the effects of MBL on scavenger receptors that may be involved both in the clearance of apoptotic cells and NTHi, as this pathogen is present at clinically important levels (>10^4^ CFU/ml BAL) in the lower airways of ∼1/3 COPD patients, and present in the bronchial tissues of 87% of patients with exacerbations compared with 33% of stable COPD patients and 0% of healthy controls [31]. These are un-encapsulated *H. influenzae* which is an important factor in predicting MBL binding. The presence of a polysaccharide capsule greatly reduces MBL binding to bacteria [32].

Our multi-parameter flow cytometric analysis of scavenger receptors on human alveolar macrophages treated with MBL detected no significant changes in surface expression of mannose receptor, Colec12 (SCARA4), Dectin, MFG, CD23 or CD36; remarkable changes were detected only for SRA. There have been extensive publications by other authors demonstrating that SRA is responsible for binding and internalization of bacteria or apoptotic cells, or dead cell-derived ligands [33]–[35]. A possible mechanism for stimulation effects of MBL on efferocytosis and phagocytosis could be an up-regulated SRA expression in macrophages. Ideally, the use of Western blot analysis to confirm the increased expression of SRA detected by flow cytometry and immunofluorescence would have been of interest; however, these were not possible due to limitations of macrophages numbers from BALs. Furthermore, our finding was in lines with a previous report on increased surface expression of SRA in rat Kupffer cells treated with recombinant MBL [23]. These data suggest that MBL facilitation of efferocytosis/phagocytosis does not necessarily involve its opsonisation effect on the target and consequently bridging the target with macrophages, but rather directly effects the cell prior to a contact with the target. Consistent with our data, SP-A, a collectin which has structural homology with MBL, was reported to stimulate alveolar macrophage phagocytosis of bacteria through increased cell surface localization of SRA [36]. The precise mechanism for MBL to induce increased expression of SRA remains yet to be defined. In recent years, immunomodulatory effects of MBL on cytokine production have been reported, and direct interaction between MBL and the TLR2/TLR4 pathway could be a possible mechanism [37]–[40]. Of relevance to our findings, a cross-talk between SRA and TLR4 pathway has also been documented [41], [42]. Thus the effects of MBL on SRA expression and efferocytosis/phagocytosis, together with other immunomodulatory effects could represent novel functions of this molecule.

There is a homology in both structure and function between human and mouse MBL. We have already shown that nebulised administration of human pdMBL at clinically achievable concentrations significantly reduced inflammation and improved efferocytosis in a smoking mouse model, restoring pulmonary macrophage efferocytic ability and reducing airway inflammation (evidenced by a significant reduction in WCC and macrophage numbers to near-normal levels) [12]. In humans, pdMBL has been designated safe in phase I and II clinical trials [43], [44]. No adverse effects were observed, nor were autoantibodies to MBL present in samples taken at 24 weeks after the last infusion. These studies support the application of pdMBL as a useful adjunct therapeutic strategy for COPD. However, the data from the present investigation needs to be considered before progressing MBL therapy to human situations. Our data strongly support the notion that oxidative stress in the airways of smokers and patients of COPD is a detrimental factor to both the structure and functions of MBL in facilitation of efferocytosis of apoptotic cells and phagocytosis of pathogens. Such effects should be taken into consideration when designing novel therapeutic approaches for COPD and possibly other chronic lung diseases where MBL may play a pathophysiological role.
